# Pharmacology of Corticosteroids for Diabetic Macular Edema

**DOI:** 10.1167/iovs.17-22259

**Published:** 2018-05

**Authors:** Scott M. Whitcup, John A. Cidlowski, Karl G. Csaky, Jayakrishna Ambati

**Affiliations:** 1 Jules Stein Eye Institute, David Geffen School of Medicine at UCLA, Los Angeles, California, United States; 2 Department of Health and Human Services, National Institute of Environmental Health Sciences, National Institutes of Health, Research Triangular Park, North Carolina, United States; 3 Retina Foundation of the Southwest, Dallas, Texas, United States; 4 Center for Advanced Vision Science, Department of Ophthalmology, Department of Pathology, Department of Microbiology, Immunology, and Cancer Biology, University of Virginia School of Medicine, Charlottesville, Virginia, United States

**Keywords:** corticosteroids, drug differentiation, intravitreal drug delivery, retinal disease

## Abstract

**Purpose:**

Corticosteroids remain the mainstay of treatment for inflammatory diseases almost 80 years after their first clinical use. Topical ophthalmic formulations of corticosteroids have been available to treat disease of the anterior segment of the eye, but the approval of corticosteroids to treat vitreoretinal diseases, including vein occlusion, diabetic macular edema, and uveitis, has occurred only recently. Although most diseases respond to corticosteroid therapy, some patients are resistant to this therapy and side effects, including cataract and elevated intraocular pressure, can limit their use. The purpose of this review is to detail the basic science of corticosteroids focusing on differences in potency, drug delivery, pharmacokinetics, and gene activation, and how these differences affect safety and efficacy in the treatment of diabetic macular edema.

**Methods:**

A review was conducted of basic science and pharmacology of the corticosteroids used to treat diabetic macular edema.

**Results:**

Clinically available corticosteroids not only have differing potency and pharmacokinetics, but also activate different genes in different target tissues. These differences are associated with distinct efficacy, pharmacokinetic, and safety profiles. It is important to understand these differences in selecting corticosteroids to treat diabetic macular edema.

**Conclusions:**

Recent advances in our understanding of the basic science of corticosteroids can explain clinical differences in these agents regarding efficacy and safety. Importantly, this understanding should allow the future discovery of additional novel corticosteroids to treat diabetic macular edema.

Corticosteroids originally were defined as a group of steroids produced by the adrenal cortex. There are two classes of adrenocortical steroids: glucocorticoids that regulate the use of carbohydrates, proteins, and fats in the body, and mineralocorticoids that regulate salt and water balance. Cortisol and cortisone are examples of glucocorticoids and aldosterone is an example of a mineralocorticoid. The first use of corticosteroids to treat disease dates to the 1940s.^1^ On September 21, 1948, Phillip Hench treated a patient with rheumatoid arthritis with a gluteal injection of 17-hydroxy-11-dehydrocorticosterone (which he called Compound E), and noted dramatic resolution of the signs and symptoms of the disease.^1^ Hench originally was led to investigate this compound following his observation that rheumatoid arthritis tended to improve during pregnancy and in patients with jaundice, both conditions associated with elevated corticosteroid levels. Similar results were observed in 13 additional patients, and on April 11, 1949, Hench et al.^2^ published these findings in a landmark article in the Proceedings of the Staff Meetings of the Mayo Clinic entitled “The effect of a hormone of the adrenal cortex (17-hydroxy-11-dehydrocorticosterone: compound E) and of pituitary adrenocorticotropic hormone on rheumatoid arthritis.” In 1950, Hench, Kendall, and Reichstein were awarded the Nobel Prize for this work.

Based on these findings, physicians began using corticosteroids for a plethora of inflammatory diseases and, in the early 1950s, ophthalmologists began using corticosteroids to treat uveitis. Interestingly, ocular inflammatory disease had been treated previously by elevating body temperature. As was the case with rheumatoid arthritis in patients with jaundice, this effect may have been a result of the induction of endogenous corticosteroid production. Sir Stewart Duke-Elder wrote, “Induced hyperpyrexia, wherein the temperature of the patient is raised to 40 or 41 degrees Celsius for a period of 4 to 6 hours can produce a dramatic effect.”^3^ He also stated that “the treatment, however, is somewhat dangerous.”^3^

Over the last 60 years, corticosteroid research has led to the syntheses of hundreds of new corticosteroids. Some of these have been developed for medical use, and a number have been used in the eye. Topical use still predominates as the most common route of ophthalmic administration of corticosteroids; but subconjunctival, sub-Tenon's, and peribulbar injections also are used frequently. In the treatment of retinal disease, most commonly posterior uveitis and macular edema, intravitreal injections are used to increase drug concentrations at target tissues and thus treatment efficacy.

Although topical formulations of corticosteroids have been approved for ophthalmic use for decades, the commercial availability of corticosteroids to treat vitreoretinal disease has occurred over the last decade. It is important to note that not all patients respond equally to all corticosteroids, and side effects can limit their use. Table 1 lists the adverse effects of corticosteroids.^4–6^ The local delivery of small amounts of corticosteroids has limited the occurrence of systemic side effects; however, local side effects, predominantly cataract and elevated IOP, still occur. The relationship between the basic science of corticosteroids and the safety and efficacy of these therapeutic agents is detailed in this review.

**Table 1:**
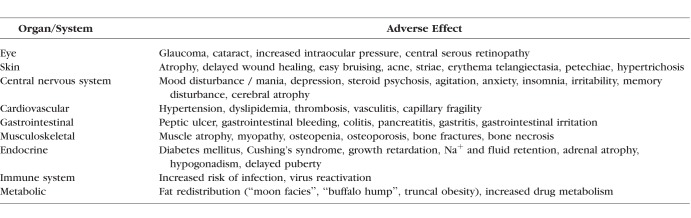
Corticosteroid Adverse Effects^4–6^

The naturally occurring corticosteroids cortisol, cortisone, and corticosterone are made in the adrenal gland and bind to the glucocorticoid and mineralocorticoid receptors in the body. At this time, three synthetic corticosteroids are used commonly to treat vitreoretinal disease: dexamethasone (DEX), fluocinolone acetonide (FA), and triamcinolone acetonide (TA). Unlike the naturally occurring corticosteroids, these molecules bind selectively to the glucocorticoid receptor and have minimal mineralocorticoid activity (Table 2).^7^ Although these three corticosteroids share biological properties, differences in structure (Fig. 1), receptor binding affinity, dose, formulation, and delivery method lead to substantial differences in pharmacokinetic profiles and gene regulation, and clinically meaningful functional differences. Understanding the differences among these drugs will be crucial to selecting the appropriate corticosteroid and route of administration to optimize patient benefit and minimize adverse effects. This review will focus on the differences between the corticosteroid treatments that have been used most commonly to treat retinal diseases and highlight those differences that can impact clinical use.

**Table 2:**
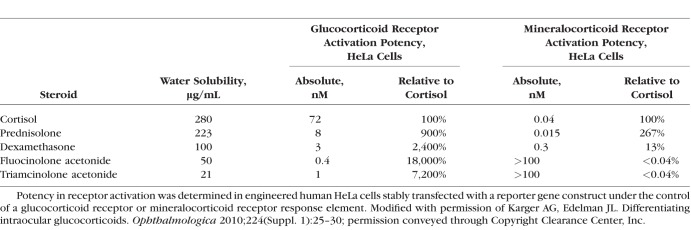
Water Solubility and Relative Activity of Selected Corticosteroids in an Immortalized Human Cell Line

**Figure 1:**
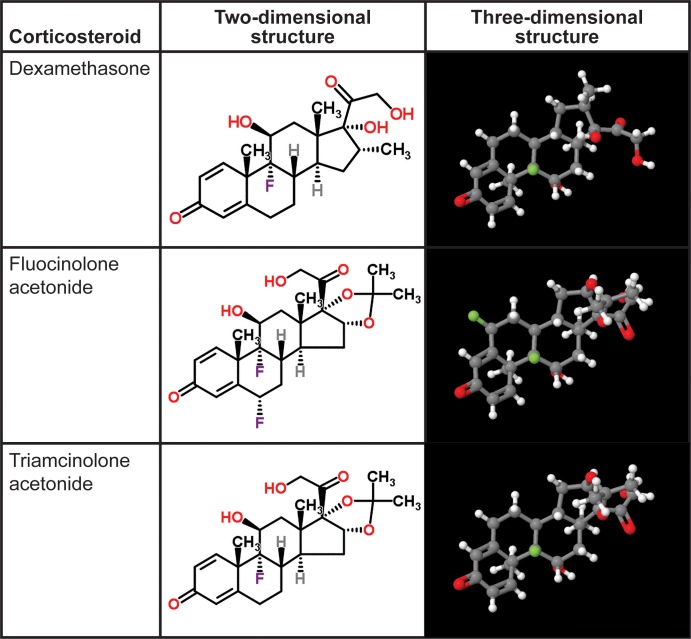
Molecular structures of corticosteroids used in the treatment of vitreoretinal diseases (from http://www.chemspider.com/Chemical-Structure.5541.html, http://www.chemspider.com/Chemical-Structure.5980.html, and http://www.chemspider.com/Chemical-Structure.6196.html; available in the public domain. Accessed March 24, 2016).

## Molecular Structure

Adrenocortical steroids have 21 carbons and a ring structure similar to cholesterol, from which the naturally occurring corticosteroids are synthesized. The two- and three-dimensional structures of DEX, FA, and TA are shown in Figure 1. DEX has a methyl group at the C16 position and a hydroxyl group at the C17 position, whereas FA and TA have an acetonide group at the C16 and C17 positions. All three molecules have a fluorine at the C9 position, but FA has an additional fluorine at the C6 position. Although the two-dimensional structures look similar, these molecules have very different three-dimensional conformations and this affects receptor binding, solubility, and pharmacokinetics. As will be shown, each corticosteroid has a unique profile of gene regulation and this leads to different clinical effects.

Corticosteroids often are assessed by comparing relative potency. However, since the biologic effects of corticosteroids are extremely complex, involving the regulation of multiple genes, and the formulation and route of administration also can affect activity, it is not possible to quantify relative potency as a single value. The assay and cell type used to assess potency also affect the results. For example, the classic table in Goodman and Gilman's textbook^8^ provides one view of the relative potencies of commonly used steroids that is very different from the relative potencies of the same steroids when assessed using different assays in an immortalized human cell line (Tables 2, 3). In the assay used to compare anti-inflammatory potency (glucocorticoid receptor mediated) in Goodman and Gilman's textbook, DEX was 5-fold more potent than TA, and both drugs had nonmeasurable mineralocorticoid activity (Table 3).^8^ In contrast, in assays using engineered human HeLa cells, TA was 3-fold more potent than DEX in glucocorticoid receptor activation and was less potent than DEX in mineralocorticoid receptor activation (Table 2).^7^ There are multiple reasons to explain these differences, including variability in receptor expression in different cell types that can impact binding based on the stereochemistry of each drug.

**Table 3:**
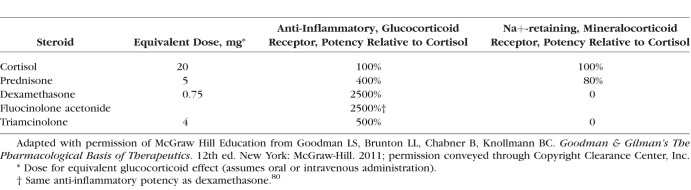
Relative Potency of Corticosteroids

## Solubility, Dose, and Drug Delivery Considerations

Water solubility is one of the main factors affecting corticosteroid pharmacokinetics. Higher water solubility can improve drug loading and bioavailability, but decreases the half-life of the drug in the vitreous.

DEX is the most water soluble of the three commonly used ophthalmic corticosteroids (Table 2) and, therefore, requires a sustained-release delivery system to maintain prolonged drug levels in the vitreous. DEX is available commercially as a sustained-release biodegradable implant (Ozurdex, Allergan plc, Dublin, Ireland) composed of poly(lactic acid-co-glycolic acid) polymers that degrade into carbon dioxide and water as DEX is released. The implant contains 700 μg DEX and is packaged in a single-use applicator with a 22-gauge needle for intravitreal injection.

FA is approximately 50% less water soluble than DEX (Table 2), but also requires a sustained-release delivery system to maintain an adequate intravitreal concentration over time. There are two commercially available FA formulations for intravitreal use: a 0.59 mg nonbioerodable implant that must be placed surgically and sutured at the pars plana (Retisert, Bausch & Lomb, Bridgewater, NJ, USA), and a 0.19 mg nonbioerodable implant that is packaged in a single-use applicator with a 25-gauge needle for intravitreal injection (Iluvien, Alimera Sciences, Inc., Alpharetta, GA, USA).

TA is the least water soluble of the three corticosteroids (Table 2). It is available commercially in the United States as Triesence (Alcon, Fort Worth, TX, USA) and Kenalog-40 (Bristol-Myers Squibb, New York, NY, USA). TA is a crystalline powder and both commercially available products are injectable suspensions containing 40 mg/mL TA in an isotonic saline solution. It is important to note that Kenalog-40 is not approved for intraocular use, but it sometimes is used off-label to treat retinal diseases. Commonly used doses of intravitreal TA in the treatment of retinal disease are 1 to 4 mg, usually delivered through a 30-gauge needle. The lower water solubility and crystalline form of TA are thought to contribute to a longer duration of effect in the vitreous (without the need for a sustained-release delivery system), but also limit the maximum dose that can be administered due to the risk of drug precipitation at higher doses. This also limits the maximum duration of drug release, because the duration of drug release of TA in the vitreous is dependent predominantly on the dose.

## Pharmacokinetics

In addition to differential gene regulation by different corticosteroids, we know that the ocular effects of corticosteroids also depend on potency, dose, and the availability of drug at the target tissue over time. The intraocular pharmacokinetic properties of the three corticosteroids used for retinal diseases are very different and impact efficacy and adverse events. Unfortunately, there are few comparative studies of these corticosteroids and, because ocular pharmacokinetics can differ depending on experimental design (including methods of measurement and the species of animal tested), the results from different studies must be compared with caution. It also should be noted that few pharmacokinetic studies of intravitreal corticosteroids have been conducted in humans, and these frequently measured drug levels in the aqueous rather than the vitreous. Although concentrations of TA were significantly lower in the anterior than the posterior chambers following intravitreal dosing in rabbits, the time course followed the same general pattern in both chambers.^9^ The aqueous pharmacokinetics of intravitreal corticosteroids in humans also appear to follow the same general pattern as the intravitreal pharmacokinetics seen in animal studies.^10^ However, drug levels in the vitreous and retina would be preferable in assessing the pharmacokinetic profile of drugs used to treat retinal diseases.

**Figure 2:**
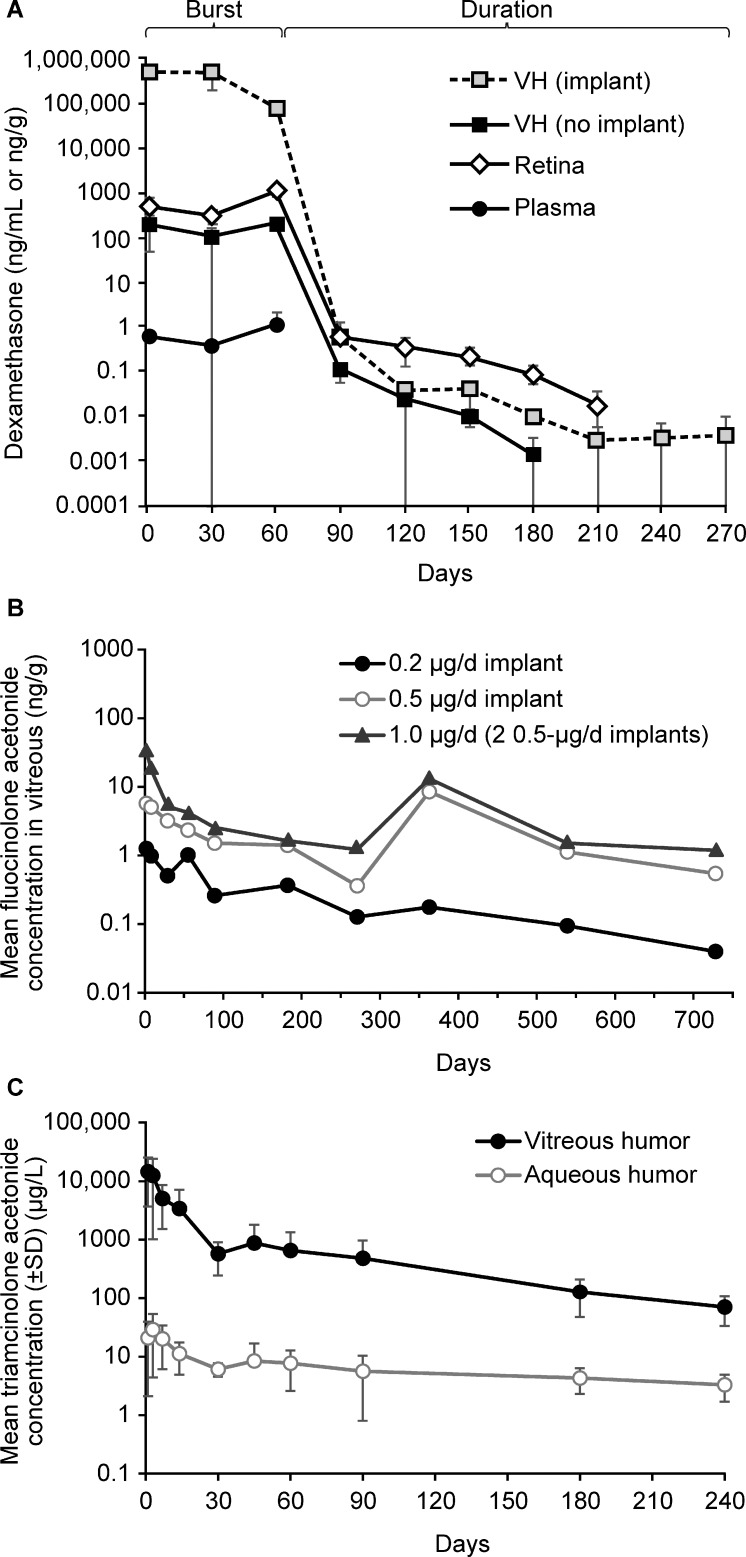
Intravitreal pharmacokinetics of corticosteroids. (A) Pharmacokinetics of dexamethasone implant in monkeys. Reprinted from Chang-Lin J-E, Attar M, Acheampong AA, et al. Pharmacokinetics and pharmacodynamics of a sustained-release dexamethasone intravitreal implant. Invest Ophthalmol Vis Sci. 2011;52:80–86. © 2011 Association for Research in Vision and Ophthalmology. (B) Pharmacokinetics of fluocinolone acetonide 0.19 mg implant in rabbits (data from Kane et al.^14^). (C) Pharmacokinetics of triamcinolone acetonide 6 mg in rabbits (data from Kamppeter et al.^9^). VH, vitreous humor with or without implant in the sample.

The intravitreal pharmacokinetics of the DEX implant has been studied in animal models in monkeys and rabbits. Following bilateral implantation in 34 male monkeys, there was a high rate of initial drug release during the first 2 months, followed by a prolonged lower level of release, with intravitreal DEX levels falling below the level of detection at 6 months (Fig. 2A).^11^ The maximum concentration (C_max_) for the retina was 1110 ng/g at day 60. The biological effect of DEX was assessed by measuring cytochrome P450 3A8 gene expression in the retina (using real-time reverse transcription-polymerase chain reaction) and exhibited a 3-fold upregulation for up to 6 months after treatment with DEX implant.^11^ The prolonged effect on gene expression may be explained by the potency of corticosteroids, such that even small, nondetectable levels of drug can have biologic effects, or by a continued effect on expression in the absence of drug. The clinical relevance of this is that efficacy may last longer than the pharmacokinetics would suggest, and adverse effects, such as increased IOP, may continue after detectable levels of corticosteroid have cleared from the eye.

The pharmacokinetics of the DEX implant also were evaluated in vitrectomized and nonvitrectomized eyes in rabbits.^12^ Drug exposure (AUC_0-t last_) with DEX was not significantly different in nonvitrectomized and vitrectomized eyes when measured in either the vitreous humor (13,600 vs. 15,000 ng/day/mL; *P* = 0.73) or retina (67,600 vs. 50,200 ng/day/mL; *P* = 0.47).^12^

There are no published human intraocular pharmacokinetic data for the DEX implant. In clinical studies of the DEX implant in patients with retinal vein occlusion or diabetic macular edema (DME), plasma levels of DEX were below the lower limit of quantitation (50 pg/mL) in most samples collected 1 to 90 days after implantation, but detectable levels of 52 to 102 pg/mL DEX were observed in 12% of samples.^13^

The intravitreal pharmacokinetic profiles of both types of FA implant have been studied in rabbits.^14,15^ Following insertion of 0.2, 0.5, or 1.0 μg/day FA implants, the vitreous humor concentration peaked at day 2 at 1.26 ng/g for the 0.2 μg/day implant, 5.75 ng/g for the 0.5 μg/day implant, and 35.9 ng/g for the 1.0 μg/day implant (Fig. 2B).^14^ Vitreous levels of FA decreased over the first 3 months with measured concentrations of 0.261 and 1.52 ng/g at day 89 for the 0.2 and 0.5 μg/day implants, respectively.^14^ Detectable levels of FA in the vitreous were found for all three doses at day 728. In a separate study of 0.5 and 2.0 mg FA implants in pigmented rabbits, vitreous concentrations ranged from 11 to 18 ng/g for the 0.5 mg implant and 75 to 146 ng/g for the 2.0 mg implant.^15^ Both types of FA implant exhibited near zero-order release kinetics with substantially lower peak vitreous concentrations than found with either TA or the DEX implant. Although efficacy and side effects also are dependent on drug potency and solubility, in general, zero-order kinetics are easier to predict, and some patients may benefit from consistently lower corticosteroid levels over a longer period, as some side effects may be exacerbated by elevated amounts of corticosteroids, even for brief periods. Other patients with other diseases may benefit from higher levels of corticosteroids for a short period; however, no data from randomized clinical trials have demonstrated these differences, and the preferential use of different corticosteroids has been based mostly on small case series.

Although to our knowledge no studies have compared the pharmacokinetics of FA 0.19 and 0.59 mg implants in eyes with or without a previous vitrectomy, human data support efficacy of FA implants in patients with a previous vitrectomy. In a retrospective study of 26 eyes from 25 patients with DME and a prior vitrectomy, treatment with one 0.2 μg/d 0.19 mg FA implant resulted in visual acuity improvement and decreased foveal thickness over a mean follow-up of 255 days.^16^

**Figure 3:**
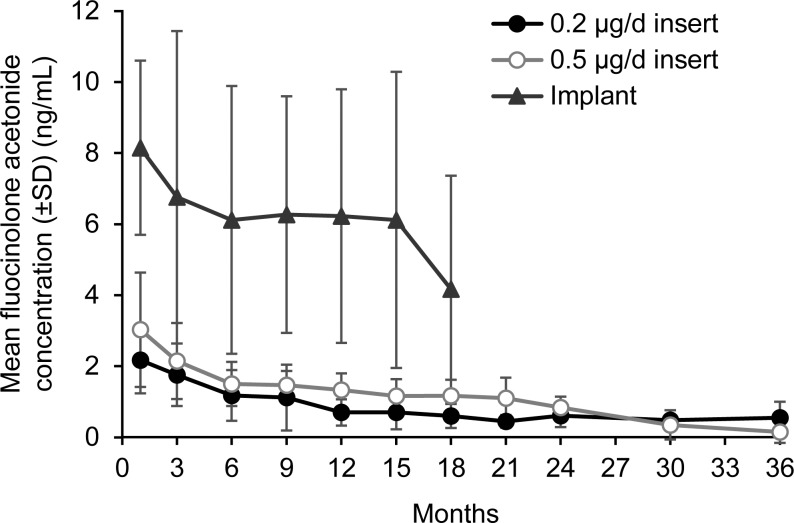
Aqueous pharmacokinetics of fluocinolone acetonide following intravitreal administration of fluocinolone acetonide insert or 0.59 mg implant in humans. Data from Campochiaro et al.^17^

The pharmacokinetics of FA following intravitreal administration of FA 0.19 or 0.59 mg implants have been studied in the aqueous humor in patients.^17,18^ In 37 patients treated with FA 0.19 mg implant for DME, mean aqueous levels of FA after 1 month were 2.17 ng/mL and 3.03 ng/mL for the 0.2 μg/day and 0.5 μg/day implants, respectively.^17^ At 36 months, mean aqueous levels were 0.15 ng/mL for the 0.5 μg/day implant (Fig. 3).^17^ A study using the FA 0.59 mg implant for treatment of uveitis found that the aqueous concentrations of FA were higher than after FA 0.19 mg implant treatment and followed the same general time course as the vitreous concentrations seen in animal studies (Fig. 3).^17^ Plasma levels of FA were below the limits of quantitation for the FA 0.19 and 0.59 mg implants.^18,19^

The pharmacokinetics of intravitreal TA have been studied extensively in animals and humans and have been summarized nicely in recent review articles.^10,20^ In a study in 18 New Zealand rabbits, vitreous TA levels were measured following intravitreal injections of 6 mg TA (Volon A, a TA formulation available in Austria and Germany; Bristol-Myers Squibb, New York, NY, USA). Vitreous TA levels were 14,434 ± 10,768, 571.3 ± 329.6, and 70.7 ± 37.0 μg/L at day 1, day 2 and month 8, respectively (Fig. 2C).^9^ The authors concluded that the decrease in concentration of TA after intravitreal injection followed a two-compartment model, with an exponential decrease in the first 4 weeks followed by a more linear decrease.

The pharmacokinetics of TA following intravitreal injection have been examined by sampling aqueous humor in humans. As was seen in rabbits, the decrease in TA after intravitreal injection in humans followed a two-compartment model. Following intravitreal injection of 4 mg TA in five eyes, peak aqueous humor concentrations ranged from 2151 to 7202 ng/mL. Mean elimination half-life was 18.6 days in the four intact (nonvitrectomized) eyes.^21^ Pharmacokinetic-pharmacodynamic modeling of intravitreal TA in patients with DME predicted a mean estimated half-life for TA of 15.4 ± 1.9 days.^22^

The vitreous and aqueous pharmacokinetics of TA are different in vitrectomized and nonvitrectomized eyes in animal studies and patients with retinal disease. At 30 days after intravitreal injection of 0.3 mg TA in rabbits, TA was detected in four of six nonvitrectomized eyes but only one of six vitrectomized eyes.^23^ In the eye of a patient who previously had undergone vitrectomy before receiving an intravitreal injection of 4 mg TA, the mean elimination half-life of TA in the aqueous was only 3.2 days compared to 18.6 days in nonvitrectomized eyes (as mentioned above).^21^

As noted earlier, in evaluating the pharmacokinetic results of intravitreal TA injections, it is important to note the specific formulation used. A recent study of four different formulations of TA for intravitreal injection found that the pharmacokinetic profile as well as the efficacy and durability of effect varied depending on the formulation used.^24^ The authors suggested that the pharmacokinetic properties appeared to correlate with TA particle size; however, other differences in the formulations could have a role.^24^

In examining the pharmacokinetic profile of drugs it is important to understand peak drug levels as well as the amount of drug delivered over time. One major difference between the DEX implant and TA and the FA implants is the extremely high doses of corticosteroid delivered to the vitreous and retina over the first 2 months of therapy. In published rabbit pharmacokinetics studies, vitreous C_max_ was 138 ng/mL for TA following a 1.2 mg injection or 460 ng/mL (estimated) with a standard 4 mg injection,^25^ the DEX C_max_ was 1300 ng/mL following DEX implant injection,^26^ and the FA C_max_ was 18 ng/g following FA 0.59 mg implant administration.^15^

Pulse administration of high-dose corticosteroids has been used successfully to treat systemic autoimmune or inflammatory diseases.^27,28^ In 1992, Beck et al.^28^ showed that high dose pulse methylprednisolone (Solu-Medrol) but not oral prednisone had a positive therapeutic effect on acute optic neuritis. Extremely high doses of corticosteroids can exert unique biological effects on inflammatory cells,^29–33^ and this may explain the results seen in these early studies. For example, high dose pulse methylprednisolone can reduce transmigration of peripheral blood mononuclear cells in patients with multiple sclerosis, but only at C_max_.^33^ However, side effects also may be exacerbated by higher peak levels of corticosteroids. Studies in autoimmune diseases, such as polymyalgia rheumatica and giant cell arteritis, showed that side effects depended not only on duration of therapy, but also on dose.^34^

A goal of retinal therapy is to reduce the frequency of injections. A key difference between the pharmacodynamic profiles of the FA implants compared to either the DEX implant or TA injections is its longer duration of action. The FA implants release drug for up to 3 years (instead of the 3 to 6 months seen with the other steroid treatments) and this is associated with a reduced retreatment frequency and overall treatment burden, and may lower the risk of treatment-associated endophthalmitis. The release profile of the implant provides more sustained, but lower peak levels of corticosteroid than either the DEX implant or TA, and this may contribute to a different efficacy and side-effect profile.

## Gene Activation Profiles of Corticosteroids

More recent data demonstrate that corticosteroids bind with specific receptors and regulate the expression of thousands of genes in almost all cells in a cell type–specific manner, resulting in changes in glucose metabolism, development, growth, and inflammation. Gene expression can be either up- or downregulated by corticosteroids, leading to an increase or decrease in protein synthesis. The receptors for corticosteroids are members of the nuclear hormone receptor family that function as ligand-activated transcription factors. Corticosteroids entering the cell interact with the glucocorticoid receptor (GR), change the shape of the GR, induce GR nuclear translocation, and then activate or repress gene transcription. In addition to their classic genomic effects, glucocorticoids also have rapid effects that may be mediated by nonspecific interactions with cellular membranes or by specific interactions with cytosolic or membrane-bound glucocorticoid receptors.^35^ However, the clinical relevance of these rapid effects remains unclear.^35^

Glucocorticoids are integral to normal physiology and survival. Data from knockout experiments have shown that the GR is critical to mediate glucocorticoid effects and sustain life. GR knockout mice display a broad range of defects and die at birth,^36^ whereas mice with targeted knockout of the GR in cardiomyocytes develop cardiac hypertrophy and heart failure, and undergo premature death.^37^ Knowledge of the structure of the GR is necessary to understand how the GR can mediate differential effects of corticosteroids administered to the eye.

**Figure 4:**
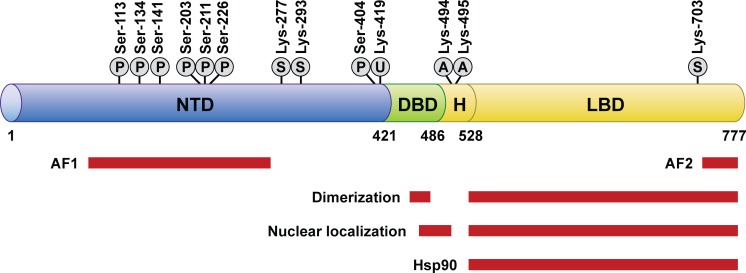
Human GR domain structure and sites of post-translational modification.^38^ Reprinted from Oakley RH, Cidlowski JA. The biology of the glucocorticoid receptor: new signaling mechanisms in health and disease. J Allergy Clin Immunol. 2013;132:1033–1044. Published by Elsevier. The regions of the receptor involved in transactivation (AF1 and AF2), dimerization, nuclear localization, and hsp90 binding are indicated, as are the sites modified by phosphorylation (P), sumoylation (S), ubiquitination (U), and acetylation (A).

Structural analysis shows that the GR is a modular protein with three domains (Fig. 4).^38^ The C-terminal domain is the ligand-binding domain where glucocorticoid binds to the receptor within a hydrophobic pocket. This domain also includes a site that binds other proteins (coregulators) only when the GR is bound by glucocorticoid. The expression of coregulators is cell-type specific, allowing the glucocorticoid to act in a cell-type specific manner. The middle domain of the GR is the DNA-binding domain, which binds the glucocorticoid response elements of genes that have regulated expression. The N-terminal transactivation domain of the GR interacts with coregulators and the transcription machinery of the cell. It also contains multiple sites for posttranslational phosphorylation, which are regulated by kinases and phosphatases in a cell-type specific manner and affect receptor metabolism and function. Between the DNA-binding domain and the ligand-binding domain is a flexible region, termed the hinge region. Nuclear localization signals are within the ligand-binding domain and at the juncture of the hinge region and DNA-binding domain.

The GR exists in a multiprotein complex in the cell cytosol until glucocorticoid binds and induces a change in the GR protein conformation. The change in conformation causes release of the chaperone proteins and exposes the nuclear localization signals, which leads to rapid translocation of the GR into the nucleus, where the GR binds to coregulators (coactivators or corepressors). The glucocorticoid- and co-regulator-bound receptor regulates gene expression by binding directly to DNA or to other DNA-bound transcription factors.^38^ X-ray crystallography studies of other members of the nuclear receptor family (estrogen, androgen, and progesterone receptors) bound to ligand have shown that the conformational change induced in the receptor varies by ligand. The conformational changes produced by the binding of different ligands expose different interacting surfaces, resulting in recruitment of different coregulators, and ultimately, a different pattern of regulation of gene expression. Crystalline structures of the GR bound to ligand are not available publicly, but there is evidence that different GR ligands induce different conformations of the ligand-binding domain of the GR, leading to differential recruitment of coregulators.^39^ This leads to further ligand-specific conformational changes in the GR and differential patterns of regulation of gene expression.

**Figure 5:**
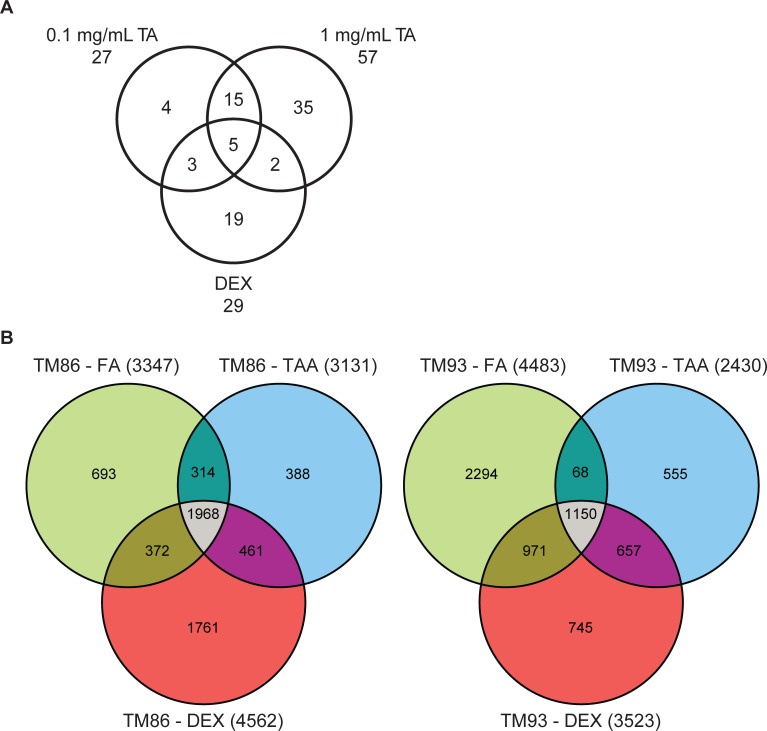
Venn diagrams showing GR ligand-specific differential gene expression in studies using microarray gene profiling. (A) Genes differentially expressed after 12 hours of exposure of human trabecular meshwork cells to 0.1 mg/mL TA, 1 mg/mL TA, or 100 nM DEX.^40^ Reprinted from Fan BJ, Wang DY, Tham CCY, Lam DSC, Pang CP. Gene expression profiles of human trabecular meshwork cells induced by triamcinolone and dexamethasone. Invest Ophthalmol Vis Sci. 2008;49:1886–1897. © 2008 Association for Research in Vision and Ophthalmology. (B) Genes differentially expressed after 24 hours of exposure of human trabecular meshwork 86 or 93 cells to 1 μM DEX, FA, or TA.^41^ Reprinted from Nehmé A, Lobenhofer EK, Stamer WD, Edelman JL. Glucocorticoids with different chemical structures but similar glucocorticoid receptor potency regulate subsets of common and unique genes in human trabecular meshwork cells. BMC Med Genomics. 2009;2:58. Published under a Creative Commons Attribution License.

The GR transcriptome regulates the expression of 2000 to 6000 genes in various cell types; a similar number of genes have upregulated and downregulated expression. Importantly, the pattern of change in gene expression produced by glucocorticoid is ligand-, concentration-, and time-dependent. A study using cultured human trabecular meshwork cells and microarray gene expression analysis demonstrated differential gene expression produced by 12 hours of exposure to 0.1 mg/mL TA, 1 mg/mL TA, or 100 nM DEX (Fig. 5A).^40^ The expression of five genes was regulated similarly by all three treatments, whereas unique changes in gene expression were seen for four genes with 0.1 mg/mL TA, 35 with 1 mg/mL TA, and 19 with 100 nM DEX. A subsequent study by Nehmé et al.^41^ evaluated the effects of 24 hours of exposure of 1 μM DEX, FA, or TA on gene expression in primary trabecular meshwork cultures from two individuals (TM 86 and TM 93). Whole genome oligo microarrays were used for gene expression profiling in this study, and the concentrations of steroid used saturated the GR, so that differential effects on gene expression could be determined to be ligand-dependent, rather than concentration-dependent. In the TM 86 and TM 93 cells, the expression of more than 1000 genes was shown to be similarly regulated by DEX, FA, and TA, but each corticosteroid also regulated the expression of a unique set of genes, and the number of genes with unique, differential expression was higher than the number of genes with common expression (Fig. 5B).

**Figure 6:**
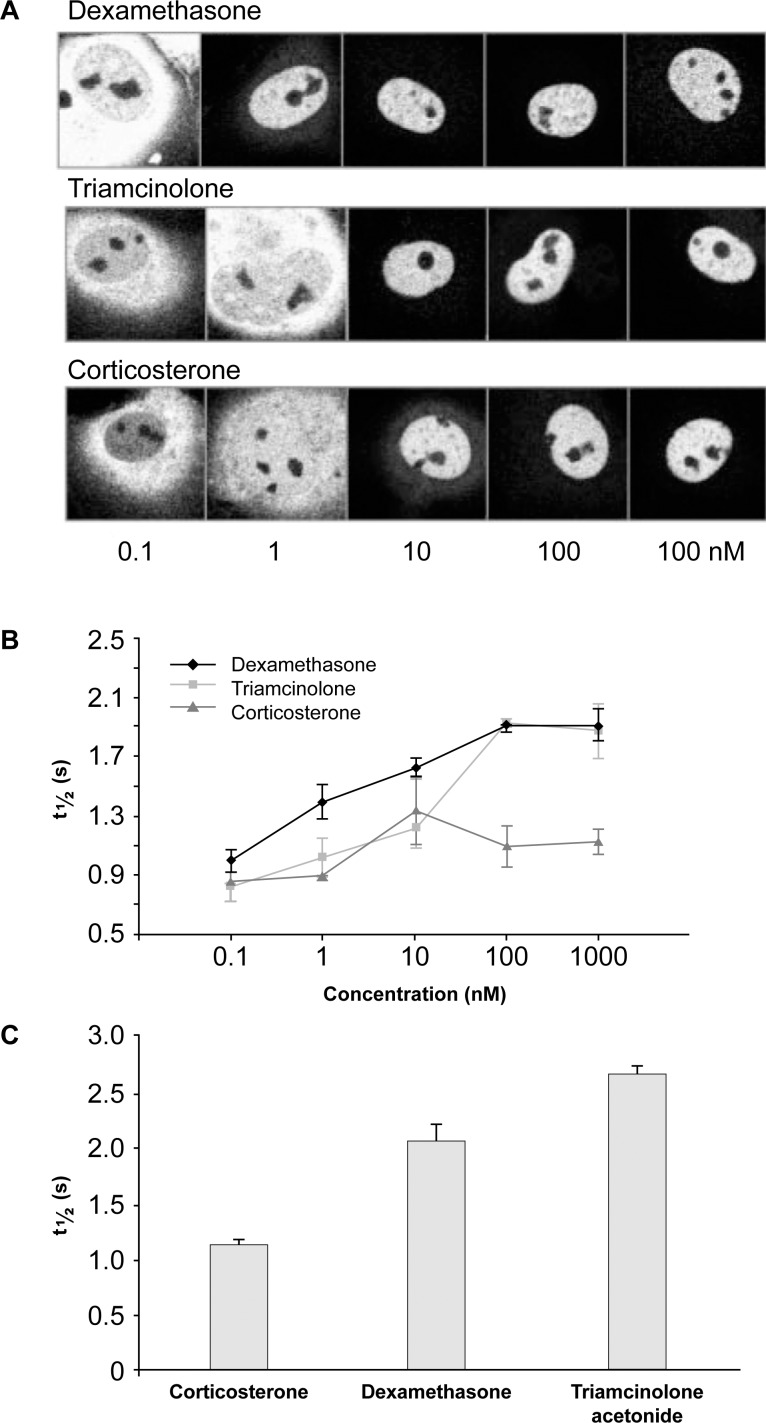
Mobility of ligand-bound human GR tagged with yellow fluorescent protein in transiently transfected COS-1 cells. (A) Translocation of bound GR into the nucleus is dependent on the steroid and its concentration. (B) The mobility of ligand-bound GR within the nucleus as measured with the recovery of fluorescence after photobleaching also is dependent on the steroid and its concentration. (C) The GR demonstrates different mobility in cells exposed to 1 μm DEX versus 1 μm TA or corticosterone. Adapted from Schaaf MJM, Cidlowski JA. Molecular determinants of glucocorticoid receptor mobility in living cells: the importance of ligand affinity. Mol Cell Biol. 2003;23:1922–1934. © 2003 American Society for Microbiology.

There appear to be multiple mechanisms by which different corticosteroids induce different patterns of gene expression. Nuclear translocation of GR bound to ligand has been visualized in cells expressing GR tagged with yellow fluorescent protein (YFP; Fig. 6A).^42^ Mobility of the ligand-bound GR-YFP was evaluated using the technique of fluorescence recovery after photobleaching. Fluorescein disappears from the bleached area of the nucleus, then reappears as nonbleached ligand-bound GR-YFP moves into the area. At concentrations below saturation, DEX was more effective than triamcinolone in causing GR-YPF to move into the nucleus, because of its higher affinity binding (Fig. 6B). The mobility of GR within the nucleus was decreased when bound to high-affinity agonist, and the time required for recovery of fluorescence after photobleaching depended upon the corticosteroid used and its concentration (Fig. 6B). At saturating concentrations, all receptors moved into the nucleus, but the mobility of the receptors within the nucleus was decreased more with TA than with DEX (Fig. 6C). With imaging technology now available, we know that the ligand-bound receptor does not form a long-lasting complex with the DNA, but instead repeatedly contacts and leaves the DNA within a few seconds, in a characteristic pattern of foci of genomic GR binding that varies by ligand.^43^ Triamcinolone, TA, DEX, cortisol, cortexolone, and corticosterone produce different patterns of foci. Transient association of ligand-bound receptor with foci has been correlated with decreased receptor mobility,^43^ and the foci could potentially represent hotspots for gene activation. Although the relationships between receptor mobility, foci, and gene expression require further study, differences in receptor mobility and foci are likely to underlie functional differences in gene expression after receptor activation by TA and DEX. For clinical and patient benefit, several pharmaceutical companies are continuing to develop new ligands for the glucocorticoid receptors that retain anti-inflammatory actions with reduced side effects.

## Corticosteroids in the Eye: Basic Science

In addition to supporting life and normal physiology, corticosteroids are critical for controlling inflammation. Inflammation has an important role in the pathogenesis of some of the most common vitreoretinal disorders, including age-related macular degeneration, branch and central retinal vein occlusion, diabetic retinopathy, and of course uveitis. One of the principal clinical manifestations of inflammation in the retina is macular edema. This edema can be present either inside cells, primarily Müller glia and retinal neurons, or be located in the interstitial space. While angiographic studies typically show extracellular leakage through the inner (retinal vascular) or outer (retinal pigment epithelium) blood–retinal barriers, electron microscopy of human donor eyes has revealed that the bulk of the macular edema actually is located intracellularly.^44,45^

A long list of inflammatory substances has been identified as important inciting factors in animal models of retinal vascular leakage and edema. Listed as broad categories, they include prostaglandins, leukotrienes, enzymes, and many cytokines (Table 4). Leading the list of cytokines is VEGF-A; however, numerous molecules in addition to VEGF-A are key drivers of retinal inflammation.

**Table 4:**
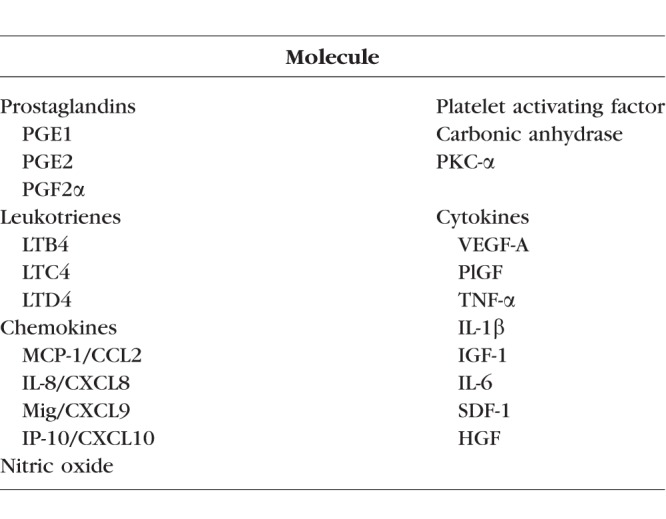
Inflammatory Substances Involved in Retinal Vascular Leakage and Edema

Many of these molecules implicated in animal models of retinal inflammation also are detectable in the aqueous or vitreous humor of patients with macular edema due to various etiologies. Again, VEGF-A levels are elevated in patients with macular edema due to many causes; however, what is important to note is that many other molecules also are elevated in these ocular compartments. In particular, several interleukins and chemokines that are potent inflammatory substances are elevated in the ocular fluids of patients with macular edema.

There is good correlation between the increasing abundance of inflammatory markers, such as IL-1B, IL-6, IL-8, MCP-1, and IP-10, and the Early Treatment Diabetic Retinopathy Study (ETDRS) severity score of diabetic retinopathy.^46,47^ In contrast, VEGF-A levels are quite elevated in the aqueous humor even in diabetic eyes with little retinopathy, and rise modestly with increasing disease severity, before the onset of macular edema.^47^ However, macular edema is accompanied by substantial elevation of IL-6 and MCP-1 as well as VEGF-A. Systemic elevation in the levels of inflammatory serum biomarkers, such as soluble E-selectin and PAI-1, are associated with an increased risk of a three-step increase in the ETDRS severity score and progression to severe nonproliferative diabetic retinopathy.^48^

Altered levels of various biomarkers in the aqueous humor, vitreous humor, and serum of diabetic patients are highly suggestive of inflammatory mechanisms driving disease severity. Recent studies using fluorescence lifetime imaging microscopy (FLIM) have provided the first evidence of ongoing inflammation in the living human diabetic retina. As opposed to techniques, such as sodium fluorescein angiography, that measure fluorescence intensity, FLIM measures the exponential decay rate (lifetime) of fluorescence. FLIM of endogenous fluorophores, such as free and protein-bound nicotinamide adenine dinucleotide (NADH), has been deployed successfully to visualize inflammation in skin diseases.^49^ Recently, time-resolve autofluorescence using FLIM ophthalmoscopy of normal and diabetic human retinas has revealed fluorescent signals consistent with tissue inflammation in this disease state.^50^

One of the important cellular aspects of inflammation is a process known as leukocyte stasis or leukostasis. This is a tightly orchestrated process whereby leukocytes are slowed down and stick to the vessel walls before they cross the vessel to enter the tissue. It involves the coordinated action of numerous groups of molecules called selectins and integrins. Of note, corticosteroids can block the various steps involved in leukostasis including downregulating the selectins and integrins.^51^

Leukostasis can be observed in various animal models, such as transient retinal ischemia induced by temporary optic nerve ligation^52^ and streptozocin-induced diabetes^53^ in rats, as well as in the human diabetic eye.^54,55^ In vivo imaging of white blood cells labeled with the fluorescent dye acridine orange that are injected into the animal can be visualized by angiography. This technique enables visualization of leukocytes that have emigrated out of the blood vessels and become stuck in the retinal tissue. Animals treated with corticosteroids have far fewer adherent leukocytes in the retina, demonstrating visually how corticosteroids can dramatically reduce the inflammatory cell infiltration. This can be attributed to the corticosteroid's effect on the amount of P-selectin and intercellular adhesion molecule-1.

Intracellular signaling processes, such as arachidonic acid pathways that lead to the formation of prostaglandins and leukotrienes, which are potent inflammatory lipid mediators, also are important in inflammatory eye diseases. Corticosteroids can enter cells; thus, they are capable of inhibiting the enzyme phospholipase A2^56^ and blocking intracellular signaling events that also are important in promoting leakage and swelling.

Müller glial cells are one of the principal loci for intracellular fluid accumulation in patients with macular edema.^44,45^ In a rat retinal slice tissue culture model, Müller glial cell swelling induced by arachidonic acid or prostaglandin E2 was reduced by corticosteroids.^57^ Similarly, in slices of retinas isolated from streptozocin-induced diabetic rats, corticosteroids dramatically reduced Müller glial cell swelling.^58^ These studies demonstrate that corticosteroids are capable of reducing intracellular swelling induced by a variety of inflammatory mediators and conditions.

It also is important to consider the cellular and tissue structural aspects that underlie macular edema. The movement of water and solutes is tightly regulated by the concerted action of intracellular and intercellular processes. For example, water and ion channels regulate fluid movement in and out of cells. Various tight junction proteins help maintain the barriers to intercellular fluid flow.

The movement of water at a molecular level is quite complex, involving, among other factors, water channels known as aquaporins. There is abundant expression of the water channel aquaporin-4 in Müller glial cells.^59^ In conditions of ischemia or inflammation, aquaporin-4 levels rise, whereas corticosteroids can reduce aquaporin-4 expression in brain tissue,^60^ providing another example of the multifaceted actions of corticosteroids.

Corticosteroids have pleiotropic activity, including restoring the structural integrity of tight junctions and reducing paracellular permeability—the movement of water and solutes between cells.^61^ Their multitude of actions include blocking intracellular signaling of inflammatory lipid mediators, such as prostaglandins and leukotrienes. They inhibit numerous cytokines and chemokines, and by modulating adenosine signaling, they can reduce blood–retinal barrier permeability.^62–65^

This pleiotropic activity observed in preclinical models would suggest that corticosteroid use in humans would result in broad-spectrum reductions of inflammatory molecules, and this has been demonstrated in several human studies. In a study of patients with DME treated with TA or bevacizumab in fellow eyes, TA treatment reduced the levels of multiple cytokines and chemokines in the aqueous humor, whereas bevacizumab profoundly reduced the levels of VEGF but did not significantly alter the levels of other inflammatory molecules.^66^

## Corticosteroids in the Eye: Clinical Considerations

Clinicians must be aware that DME is driven by multiple factors, but appears in some cases to be controlled by VEGF overexpression and/or inflammatory factors. At present, there are no clear diagnostic differentiators that would allow clinicians to identify the main driver of the DME. Although some studies have demonstrated decreases in vitreous levels of factors including angiopoietin-2, hepatocyte growth factor, and endocrine gland-derived vascular endothelial growth factor, following corticosteroid therapy that correlated with decreases in retinal edema, measurement of these factors has not been used to guide therapy.^67^ As such, a therapeutic diagnostic approach may be appropriate where a therapy with a different mechanism of action is administered if initial treatment is suboptimal. For example, an initial approach may be to treat all clinically significant DME with an anti-VEGF agent. A recent analysis of a Diabetic Retinopathy Clinical Research Network DME trial revealed that the clinical response following three monthly injections of ranibizumab was predictive of long-term visual outcomes.^68^ Lazic et al.^69^ published significant improvements in central foveal thickness and visual acuity in 16 eyes of 15 patients with refractory DME following three anti-VEGF injections who then underwent injection of a corticosteroid.

Therefore, a reasonable approach is to re-evaluate a patient at 3 months and consider initiating steroid therapy if limited or poor response to anti-VEGF therapy is noted. There are two corticosteroids approved by the United States Food and Drug Administration (FDA) to treat DME. The DEX implant is indicated to treat DME^13^ and was approved by the FDA in 2014. The FA 0.19 mg implant also was approved by the FDA in 2014 to treat DME in patients who have been treated previously with a course of corticosteroids and did not have a clinically significant rise in IOP.^70^ TA is not approved to treat DME, but is used off-label for this indication.

There is support in the literature for the intravitreal use of all three of these corticosteroids. For example, Lazic et al.^69^ published significant improvements in central foveal thickness and visual acuity in 16 eyes of 15 patients with refractory DME following three anti-VEGF injections who then underwent injection of DEX implant. In addition, Kim et al.^71^ reported on 40 eyes of 34 patients with persistent DME despite undergoing previous bevacizumab injections that received 20 mg of posterior subtenon TA. At 2 months, the mean central retinal thickness had decreased by 108 μm while the logMAR vision had improved by 0.06. Finally, Schmit-Eilenberger^72^ reported on 15 eyes of 10 patients with persistent DME after treatment with anti-VEGF and corticosteroid (TA or TA and DEX implant) that subsequently underwent injection of one 0.2 μg/d FA implant. BCVA improved in 73.3% of the eyes and central foveal thickness decreased on average by 206.3 μm.

In choosing therapy for DME, side effects also must be considered. Cataract and increased IOP are the most commonly reported adverse effects of corticosteroids in the treatment of DME. In the two randomized, 3-year, sham-controlled studies of DEX implant in DME, among patients with phakic study eyes, cataract was reported in 68% of DEX implant-treated patients compared to 21% of sham-treated patients.^13^ IOP elevation greater than or equal to 10 mm Hg from baseline was reported in 28% of DEX implant-treated patients and 4% of sham-treated patients, and use of IOP lowering medication was reported for 42% of DEX implant-treated and 10% of sham-treated patients.^13^ Surgical intervention for elevated IOP was reported for 1.2% of DEX implant-treated and 0.3% of sham-treated patients.^13^ In the two randomized, 3-year, sham-controlled studies of FA 0.19 mg implant in DME, among patients with phakic study eyes, cataract was reported in 82% of FA implant-treated and 50% of sham-treated patients.^70^ IOP elevation greater than or equal to 10 mm Hg from baseline was reported in 34% of FA implant-treated and 10% of sham-treated patients, and use of IOP-lowering medication was reported in 38% and 14%, respectively.^70^ Surgical intervention for elevated IOP was reported in 5% and 1% of patients, respectively.^70^

Side effects related to administration of the corticosteroids also are reported commonly. Conjunctival hemorrhage was reported in 22% of DEX implant-treated versus 16% of sham-treated patients and in 13% of FA implant-treated versus 11% of sham-treated patients.^13,70^ There is a risk of migration of implant into the anterior chamber in patients with previous cataract surgery and absent or torn posterior lens capsule for DEX and FA implants.^13,70^

The response to treatment with intravitreal steroids may differ depending on the type of corticosteroid used, although there are no direct comparisons in well-controlled randomized clinical trials. Zucchiatti et al.^73^ presented three patients with refractory DME treated previously with other corticosteroids who demonstrated significant improvement in visual acuity and central retinal thickness after treatment with a single DEX implant. Similarly, Augustin et al.^74^ presented a subgroup of subjects from the phase 3 trial of DEX implant in DME (MEAD trial) who had received prior treatment. Schmit-Eilenberg^72^ reported a case series of patients with DME refractory to treatment with other corticosteroids who demonstrated significant visual and anatomic improvement following subsequent treatment with FA implant. Variability in response to corticosteroids may be driven not just by the direct activity of the corticosteroid, but also, by dose and pharmacokinetic properties.

## Future Research

Differential regulation of gene expression by corticosteroids is an area of active research. Drug discovery programs aim to develop novel, selective GR agonists that retain the ability to regulate gene expression needed for therapeutic effects while reducing the regulation of gene expression that leads to unintended or adverse effects.^75,76^ To achieve this aim, more research is needed to better understand the molecular pathways involved in glucocorticoid-mediated reduction of inflammation.

A key issue in the treatment of retinal disease is the heterogeneity of response to corticosteroids and other therapies, such as anti-VEGF agents. In the case of corticosteroids, the magnitude of response differs among patients and also may differ within patients over time. In addition, there are relatively common glucocorticoid receptor polymorphisms that confer resistance or hypersensitivity to glucocorticoid.^38^ Glucocorticoid responsiveness also is likely to be affected by patient characteristics, such as sex,^77^ as well as by disease variables, such as glycemic status in DME. The patient and disease characteristics that determine response to therapy are not well understood, and these require further investigation.

The problem of diminished responsiveness to corticosteroid treatment over time in some systemic disease also may be seen in retinal disease. If the reason for reduced response over time is a downregulation of GR, it is possible that it may be alleviated by pulse dosing and a drug holiday, and an implant with pulse corticosteroid release may be more beneficial than an implant that provides a low sustained concentration of corticosteroid. Furthermore, inflammasome activation, a characteristic of diabetic retinopathy,^78^ leads to reduced GR levels,^79^ which could be a potential mechanism for decreased responsiveness to corticosteroid over time. Studies are needed to determine whether inhibition of the inflammasome allows corticosteroids to have greater therapeutic effect in diabetic retinopathy.

Cell type–specific effects of glucocorticoids in ocular tissues have not been well studied, and it will be important to determine whether DEX, FA, and TA differentially regulate proteins known to be involved in retinal pathology. For example, the relative effects of DEX, FA, and TA on VEGF expression should be examined. Also, differential regulation of the expression of ion channels and aquaporins in Müller cells by these corticosteroids should be examined, because of the contribution of intracellular fluid accumulation to macular edema.

## Conclusions

Corticosteroids remain the mainstay of therapy for inflammatory diseases, including vitreoretinal diseases, such as vein occlusion, DME, and uveitis. Recent research has shown that corticosteroids differ in pharmacokinetic properties and gene activation in target tissues and that these differences affect clinical efficacy and safety. It is important to understand these basic science differences in order to select the best therapeutic agent for patients and to discover novel corticosteroids with even better safety and efficacy profiles for ophthalmic disease.
